# Serotonin-Mediated Anti-Allodynic Effect of Yokukansan on Diabetes-Induced Neuropathic Pain

**DOI:** 10.3390/jcm13144276

**Published:** 2024-07-22

**Authors:** Yoko Kajikawa, Hiroshi Yokomi, Soshi Narasaki, Satoshi Kamiya, Hirotsugu Miyoshi, Takahiro Kato, Yasuo M. Tsutsumi

**Affiliations:** Department of Anesthesiology and Critical Care, Hiroshima University Hospital, Hiroshima 734-8551, Japan

**Keywords:** yokukansan, diabetes mellitus, 5-HT receptor, neuropathic pain

## Abstract

**Background:** Diabetic neuropathic pain is a known complication of diabetes mellitus (DM) and results from the complex interaction of various factors affecting the nervous system. Yokuansan (YKS) is a versatile traditional Japanese herbal medicine with a wide range of applications, especially in pain management and neurological manifestations. YKS has analgesic properties for nerve damage and is a potential treatment for DM-induced neuropathic pain, especially in patients with diabetic neuropathy. Thus, we examined the anti-allodynic effect of YKS on DM-induced neuropathic pain. **Methods:** All experiments were performed on 6-week-old male Sprague–Dawley rats. DM and diabetic neuropathy were induced in rats with streptozotocin. Mechanical allodynia was assessed using dynamic plantar esthesiometry. Additionally, we conducted an immunological assessment of microglia cell changes in the spinal cord and an experiment to clarify the involvement of serotonin. **Results:** Diabetes significantly reduced withdrawal thresholds in rats during the initial two weeks of the experiment, which stabilized thereafter. However, this effect was not investigated in the control group. We assessed, using the dynamic plantar test, the anti-allodynic effects of orally administered YKS (1 g/kg). Daily YKS administration significantly increased the withdrawal threshold in DM animals. Additionally, oral YKS reduced the expression of Ibal-1-positive microglia. To elucidate the mechanism of action of YKS, we explored the involvement of serotonin (5-hydroxytryptamine [5-HT]) receptors in mediating its effects. Intrathecal administration of 5-HT receptor antagonists (WAY-100635, ketanserin, and ondansetron) inhibited the protective effects of YKS. **Conclusions:** YKS exhibited an anti-allodynic effect, suggesting that YKS may activate 5-HT receptors in the spinal cord, thereby alleviating diabetic neuropathic pain.

## 1. Introduction

Diabetic neuropathic pain, a complication of diabetes, results from the complex interaction of several factors affecting the nervous system [1]. Chronic hyperglycemia, a hallmark of diabetes, triggers a cascade of metabolic abnormalities that damage nerve tissue. The resulting diabetes-induced inflammation exacerbates neuropathic pain [2]. The inflammatory response fueled by hyperglycemia and other metabolic abnormalities further compromises nerve health and amplifies pain signaling [2]. In addition, neurovascular dysfunction, another consequence of diabetes, disrupts blood flow to nerves, depriving them of essential nutrients and oxygen and increasing their susceptibility to injury and pain [3,4].

Furthermore, oxidative stress in nerve tissue exacerbates the vulnerability of nerve tissue to injury and dysfunction. Oxidative stress from hyperglycemia-induced free radical production impairs cellular function, causing neuronal injury and contributing to the development of neuropathic pain. Such mechanisms are implicated in the etiology of diabetic neuropathic pain, which results in debilitating symptoms such as burning, tingling, and shooting pain in affected individuals [5,6]. Thus, diabetes-induced neuropathic pain is a complex condition that requires comprehensive management to address both the underlying causes and symptoms. Effective treatment often involves a combination of lifestyle modifications, medication, and alternative therapies tailored to individual needs and responses. Regular monitoring and collaboration with healthcare providers are crucial for optimizing management and improving the quality of life for individuals with diabetic neuropathy [5,6].

It is a global challenge to develop suitable therapeutic methods against polyneuropathy. Monomodal, multimodal, and complementary medical therapeutic approaches are recommended [7]. Japanese herbal medicine (Kampo), which originated from ancient Chinese medicine and developed rapidly from the 16th through the 19th centuries, is primarily used in traditional Japanese medicine. The universal healthcare system was established in Japan in 1961 and started covering 4 Kampo extracts as prescribable medications in 1967 [8]. More than 70% of physicians prescribe Kampo drugs in Japan and about 140 Kampo products are available under the national health insurance system today [9]. Yokukansan (YKS), one Kampo product with a long history of use, is a traditional herbal remedy consisting of a mixture of seven herbal medicines known for their sedative effects, especially in the treatment of various neuropsychiatric symptoms. It is traditionally used in Japanese medicine to treat a variety of symptoms, including behavioral and psychological symptoms of dementia [10,11]. YKS primarily affects the central nervous system and alleviates symptoms related with neuropsychiatric conditions, such as anxiety, insomnia, irritability, agitation, and dementia-related symptoms. Studies have proposed that YKS may function through mechanisms such as modulation of neurotransmitter systems and regulation of neuroinflammation. YKS has also shown analgesic effects in models of entrapment nerve injury pain, which is believed to result from its partial agonist action on serotonin receptors, activating the descending pain inhibitory system in the spinal cord [12,13,14,15,16,17,18].

Recently, we showed that YKS activates 5-hydroxytryptamine (5-HT) receptors in the spinal cord, thereby alleviating paclitaxel (PTX)-induced neuropathic pain [19]. However, the effects of YKS on diabetic neuropathic pain are unknown. Therefore, in this study, we examined the possible anti-allodynic effects of YKS on diabetic neuropathic pain. In addition, we investigated the role of serotonin, a key site of analgesic action of YKS, by intrathecal administration of a serotonin receptor antagonist.

## 2. Materials and Methods

### 2.1. Animals

Six-week-old male Sprague–Dawley rats weighing 250–300 g were purchased from Charles River Laboratories Japan Inc. (Kanagawa, Japan). The rats were stored in a controlled environment with a temperature maintained at 23 ± 2 °C and a 12-h light–dark cycle (lights on from 08:00 to 20:00), with access to food and water ad libitum. All experiments were conducted within the same time window to minimize the disruption of physiological rhythms. Ethical approval for all animal experimental procedures (approval number: A16-68 (6 September 2016)) was obtained from the Committee of Animal Experimentation, Hiroshima University. The animals were handled and cared for in accordance with the Guide for the Care and Use of Laboratory Animals.

### 2.2. Drugs

Streptozotocin (STZ) was purchased from Sigma (St. Louis, MO, USA). YKS was purchased from Tsumura & Co. (Tokyo, Japan) and consisted of 7 herbal components: Atractylodes lancea rhizome, Poria sclerotium, Cnidium rhizome, Uncaria hook, Angelica root, Bupleurum root, and Glycyrrhiza (Table 1). YKS was dissolved in distilled water in this study. 4-Chloro-DL-phenylalanine methyl ester hydrochloride (PCPA; Sigma, St. Louis, MO, USA) was used as a serotonin synthesis inhibitor. Each of the 5-hydroxytryptamine (5-HT) receptor antagonists, WAY-100635 (5-HT_1A_ receptor antagonist; Abcam, Tokyo, Japan), ketanserin (5-HT_2A/2C_ receptor antagonist; Wako, Tokyo, Japan), and ondansetron (5-HT_3_ receptor antagonist; Abcam, Tokyo, Japan), were dissolved in 10 µL of 100% dimethyl sulfoxide (DMSO; Sigma).

### 2.3. Animal Model and Animal Behavioral Test

STZ was used to induce diabetes mellitus (DM) and diabetic neuropathy in rats. The 6-week-old rats were administered STZ (75 mg/kg) intraperitoneally, and blood glucose levels were measured 1 week after STZ administration; rats with blood glucose levels above 350 mg/dl were defined as DM rats. DM-induced neuropathic pain was assessed for automechanical stimulation and allodynia using behavioral tests with a dynamic plantar esthesiometer (Ugo Basile SRL, Gemonio, Italy).

**Figure 1:**
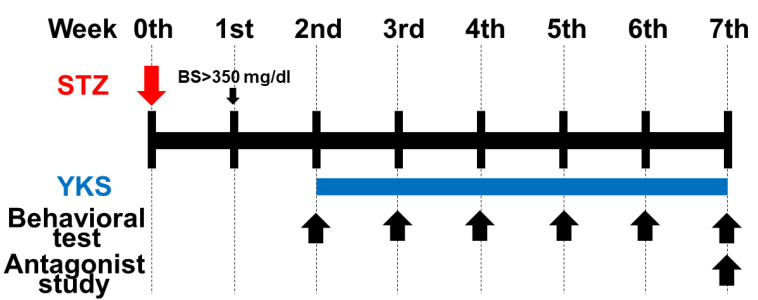
Experimental protocol. Male Sprague–Dawley rats were administered streptozotocin (STZ) intraperitoneally, and rats with blood glucose levels above 350 mg/dl after 1 week were used as diabetic rats. Yokukansan (YKS) 1 g/kg was given daily through the gastric tube for 5 weeks, from the 2nd week to the 7th week. Behavioral tests were performed weekly starting in the 2nd week (before YKS administration). In addition, a study on 5-hydroxytryptamine receptor antagonists was conducted on rats in the DM/YKS group during the 7th week after tubing surgery. STZ = streptozotocin; YKS = yokukansan; BS = blood sugar.

The DM animals were randomized into the following two groups: DM and DM/YKS groups (Figure 1). DM rats took either YKS (1 g/kg) (DM/YKS group) or distilled water (DM group) via an oral gastric tube daily for 5 weeks, starting from the 2nd week of the experiment. Paw withdrawal responses were compared between the DM and DM/YKS groups from the 2nd week before YKS administration to the 7th week, with weekly assessments. On the day of the behavioral test, YKS was administered after the test. YKS was administered in the afternoon every day. As described before [19], a movable touch-stimulator unit with a von Frey type was used for behavioral experiments. Briefly, the paw withdrawal threshold was measured six times, and four paw withdrawal threshold values were recorded, excluding the maximum and minimum values. Threshold values were expressed in grams, with a cutoff value of 50 g within 20 s.

### 2.4. Immunological Assessment of Glial Cell Changes by Immunostaining of the Spinal Cord

The immunological response and glial cell changes were evaluated in both the DM and DM/YKS groups on the 7th week of the experiment. The L4/L5 segments of the spinal cord were exposed via laminectomy from the lumbar vertebrae, and samples of the lumbar enlargement were fixed in paraformaldehyde as described previously [19]. Antibodies against Iba1 were used for labeling microglia, and GFAP antibodies were used for labeling astrocytes. GFAP antibody (rabbit monoclonal, 1:500 dilution; Abcam) and Iba1 antibody (goat polyclonal, 1:200 dilution; Abcam) were applied for 1 h for labeling astrocytes and microglia, respectively. The sections were viewed under a microscope (ECLIPSE 90i, Nikon, Tokyo, Japan), and positive cells were measured using ImageJ software (Ver. 1.54a). The expression levels of activated Ibal-1-positive microglia and GFAP-positive astrocytes were examined in the DM and DM/YKS groups by counting the number.

### 2.5. 5-HT Synthesis Inhibitors and 5-HT Receptor Antagonists

We conducted an experiment to clarify the involvement of serotonin in the DM/YKS group at 7 weeks. PCPA, which depletes 5-HT, was injected intraperitoneally into the DM/YKS group (PCPA group). A total of 100 mg/kg PCPA was dissolved in 1 mL of saline and administered for 3 days immediately prior to behavioral testing [12]. The control group was injected with saline as a vehicle. Paw withdrawal thresholds were compared between the control (n = 10) and PCPA (n = 5) groups using a dynamic plantar esthesiometer. Next, in the 7th week, we observed the effects of 5-HT receptor antagonists on the DM/YKS rats. WAY-100635 (a 5-HT1A receptor antagonist), ketanserin (a 5-HT2A/2C receptor antagonist), and ondansetron (a 5-HT3 receptor antagonist) were employed as 5-HT receptor antagonists as described before [19].

The 5-HT receptor antagonists (WAY-100635 60 μg, ketanserin 30 μg, and ondansetron 30 μg) were dissolved in 10 μL of 100% DMSO, and then DM/YKS rats were injected intrathecally with 100% DMSO (vehicle) or 5-HT receptor antagonists. Each effect was assessed by behavioral experiments 60 min after injection.

### 2.6. Statistical Analysis

The values of the experiments are expressed as mean ± S.E.M. Statistical analysis was calculated using GraphPad Prism software (Ver. 7.05. GraphPad Software Inc., San Diego, CA, USA). Comparisons between two groups were compared using the Mann–Whitney U test. The Bonferroni method was used for post-hoc analysis. Statistical significance was set at *p* < 0.05.

## 3. Results

### 3.1. Experimental Animals

Ninety-eight male rats were used. The success rate of the animal model was 76%. Animals with neurological damage or deterioration of general condition were excluded from subsequent experiments. Specifically, 4 animals from the DM group, 6 from the DM/YKS group, and 12 from the antagonist study were excluded.

### 3.2. Diabetes Effectively Decreased the Withdrawal Threshold

**Figure 2:**
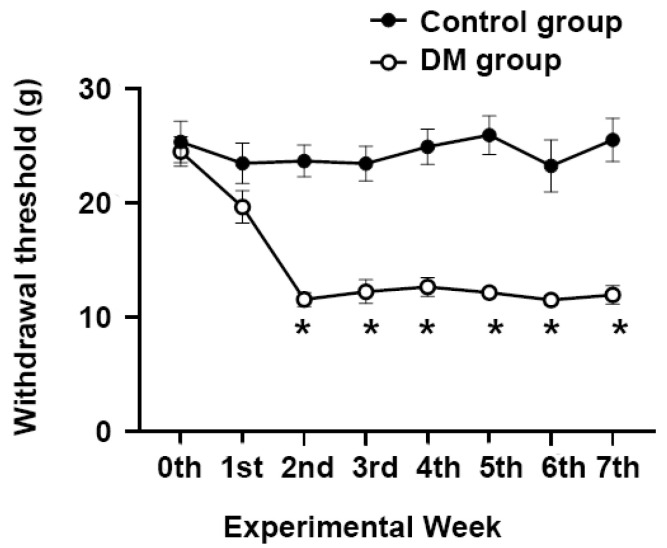
Time course of the effects of intraperitoneal administration of normal saline (control group) or streptozotocin (DM group) on the withdrawal thresholds of the hind paw using a dynamic plantar esthesiometer in SD rats. Data are presented as mean ± S.E.M. * *p* < 0.001 compared with the DM group. N = 8 in each group. DM = diabetes mellitus.

We utilized an animal model to study neuropathic pain induced by DM. DM significantly lowered the withdrawal threshold during the initial 2 weeks and subsequently stabilized (Figure 2). However, this effect was not investigated in the control group.

### 3.3. YKS Improved Allodynia

**Figure 3:**
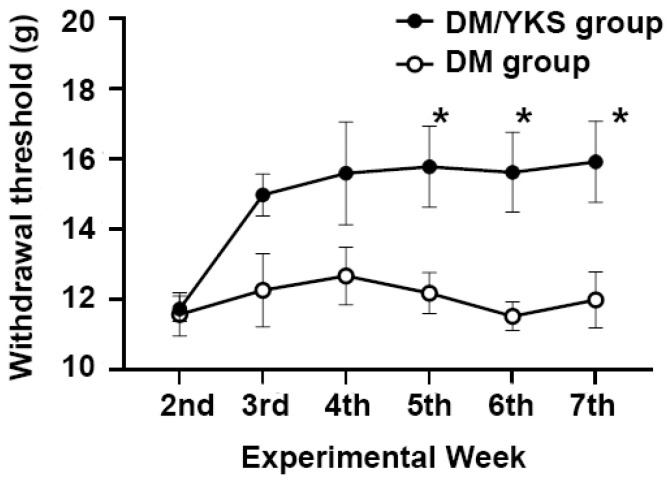
The effects of YKS on the withdrawal thresholds of the hind paw in diabetic rats. Using diabetic rats, withdrawal thresholds were measured weekly for 5 weeks in rats treated with YKS (PTX/YKS group) or distilled water (DM group), starting before YKS administration (week 2). Data are presented as mean ± S.E.M. * *p* < 0.05 compared with the DM group. N = 8 in each group. DM = diabetes mellitus; YKS = Yokukansan.

Allodynia was effectively ameliorated by YKS (Figure 3). The DM/YKS group had a significantly higher withdrawal threshold than that of the DM group on the 5th week [15.8 ± 1.2 g vs. 12.2 ± 0.6 g: *p* < 0.05]. This effect of YKS continued throughout the duration of YKS administration. There was no significant difference in body weight between the two groups.

### 3.4. YKS Prevented Microglia Activation

**Figure 4:**
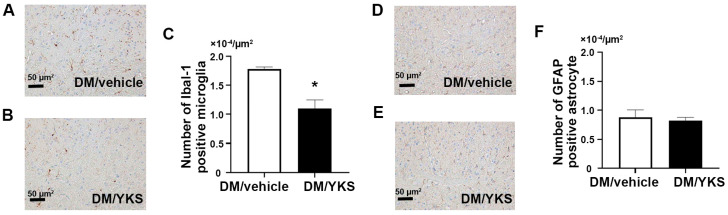
A comparison of the Ibal−1−positive microglia (dark blue stained) expression levels between diabetes/vehicle (DM/vehicle) group (**A**) and diabetes/yokukansan (DM/YKS) group (**B**). (**C**) revealed the number of Ibal−1−positive microglia. GFAP-positive (dark blue stained) astrocyte expression levels between DM/vehicle group (**D**) and DM/YKS group (**E**). (**F**) shows the number of GFAP-positive microglia. * *p* < 0.01; N = 5 in each group. DM = diabetes mellitus; YKS = Yokukansan.

Since morphologically altered and activated microglia and astrocytes are Ibal-1-positive microglia and GFAP-positive astrocytes, the immunological evaluation suggests that both microglia and astrocytes are morphologically altered. In a group of DM rats treated with YKS (DM/YKS) versus the DM group treated with distilled water, the expression level of Ibal-1-positive microglia was significantly inhibited by approximately 40% in YKS-treated rats (1.1 × 10^4^ vs. 1.7 × 10^4^ µm^2^; *p* < 0.01), Figure 4A–C), whereas there were no significant differences in GFAP-positive astrocytes between the two groups (0.8 × 10^4^ vs. 0.9 × 10^4^ µm^2^; *p* = 0.74, Figure 4D–F).

### 3.5. Effects of YKS on Serotonin in the Spinal Cord

**Figure 5:**
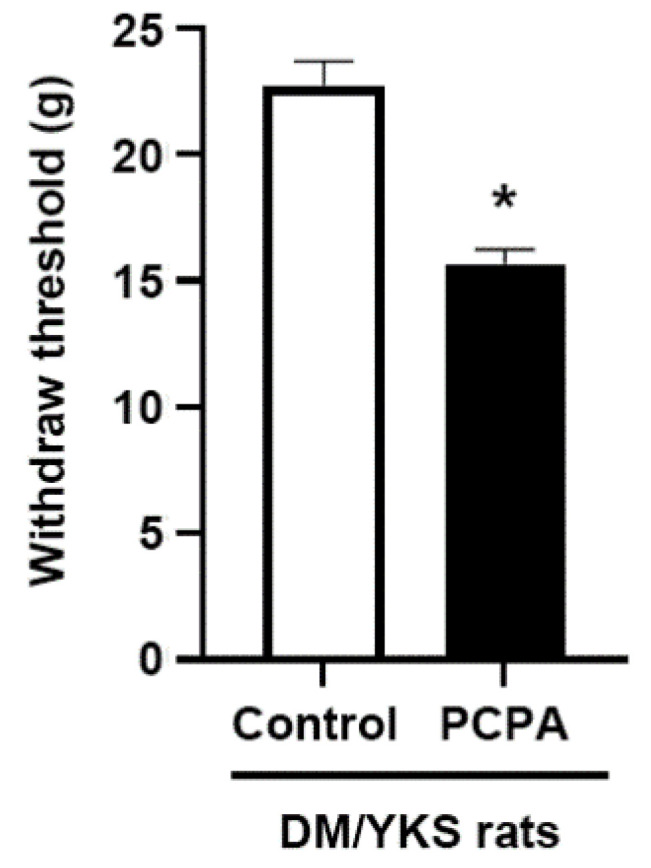
Serotonin depletion and the effect of Yokukansan (YKS). The effect of the intraperitoneal injection with 4-Chloro-DL-phenylalanine methyl ester hydrochloride (PCPA) on the anti-allodynic effect of YKS. The diabetes/YKS (DM/YKS) rats were injected intraperitoneal with PCPA (PCPA group) or a saline vehicle (control group). Data are presented as mean ± S.E.M. Control group, N = 10 and PCPA group, N = 5. * *p* < 0.001, compared with the control group. DM = diabetes mellitus.

Intraperitoneal administration of PCPA (central serotonin-depleting agent) lowered the paw withdrawal threshold compared to the controls (15.7 ± 0.6 vs. 22.7 ± 1.0 g: *p* < 0.001) (Figure 5).

### 3.6. YKS Was Mediated by 5-HT1A, 5-HT2A/2C, and 5-HT3 Receptors

**Figure 6:**
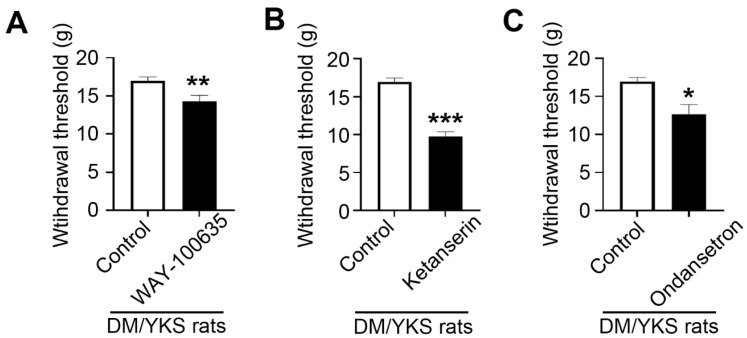
Effect of intraspinal injection of 5-hydroxytryptamine (5-HT) receptor antagonists on the anti-allodynic effect of YKS. Thresholds were measured using a dynamic plantar esthesiometer 5 weeks after YKS (DM/YKS) administration. Rats received intrathecal injections of 5-HT antagonists ((**A**) WAY100635, (**B**) ketanserin, or (**C**) ondansetron) or DMSO in the control group. The threshold was assessed after 60 min. Values are shown as mean ± S.E.M. Control group: N = 10; WAY-100635 group and ondansetron group: N = 5; ketanserin group: N = 7. * *p* < 0.05, ** *p* < 0.01, *** *p* <0.001, compared with the control group. DM = diabetes mellitus; YKS = Yokukansan.

Withdrawal thresholds were significantly lower in the groups treated with intrathecal WAY-100635, ketanserin, and ondansetron (14.3 ± 0.7 g, 9.7 ± 0.6 g, and 12.7 ± 1.3 g, respectively). The observed differences were statistically significant compared to the control group. These data suggest that YKS is mediated by 5-HT1A, 5-HT2A/2C, and 5-HT3 receptors (Figure 6).

Rats received 60 μg WAY100635 (5-HT1A receptor antagonist), 30 μg ketanserin (5-TH2A/2C receptor antagonist), and 30 μg ondansetron (5-HT3 receptor antagonist), and DMSO in the control group. Thresholds were assessed 60 min later. Data are presented as mean ± S.E.M. Control group: N = 10, WAY-100635 group and ondansetron group: N = 5 ketanserin group: N = 7. * *p* < 0.05, ** *p* < 0.01, *** *p* < 0.001 compared with the control group.

## 4. Discussion

In this study, YKS was observed to effectively decrease the withdrawal threshold in the animal model of diabetic neuropathic pain. YKS demonstrated efficacy in improving allodynia and significantly increasing the withdrawal threshold in DM models. The observed efficacy of YKS may be mediated by 5-HT receptors in the spinal cord.

YKS has been used in patients with symptoms of dizziness, irritability, neurosis, insomnia, and tardive dyskinesia and in infants with nocturnal crying and seizures. There have been reports of improvement in behavioral and psychological symptoms in patients with dementia with the use of YKS. Recent clinical and basic preclinical studies have suggested that YSK may have analgesic properties for neuropathic pain. However, few studies have investigated the analgesic effects of YSK for acute pain [20].

In addition, there is no consensus on the method of administration or dosage of YSK. Several experiments have shown that YKS can be administered to rodents based on accurate calculations of food intake. In this study, YKS was administered through a gastric tube because this method cannot be used to determine the exact dosage. Previous studies have reported anti-allodynic effects of 0.3 g/kg and 1 g/kg YKS on peripheral neuropathy in rats [12]. We chose the 1 g/kg dose of YKS based on previous studies [21,22]. Thus, this dose of YKS is quite high in humans (approximately 5–15 g/day), but animal studies suggest that this dose is acceptable.

Diabetes is a chronic condition characterized by high blood glucose levels. Diabetes can lead to neuropathy, including allodynia, because of the damage caused by high blood sugar levels in the nerves [1]. In diabetes, prolonged periods of high blood glucose levels can impair blood flow to nerve tissues, thereby reducing the supply of nutrients and oxygen. Consequently, nerve cells struggle to receive adequate nourishment to maintain their normal function. Moreover, high blood sugar levels can damage the cell walls of nerve tissues, disrupting the ability of nerve cells to transmit information effectively [3,4]. This can lead to a diminished capacity of the sensory nerves to perceive pain, resulting in symptoms such as allodynia. Neuropathy caused by diabetes is often referred to as peripheral neuropathy, where nerves in the extremities are damaged, leading to symptoms such as pain and numbness [6]. Neuropathy is a significant complication in individuals with diabetes, emphasizing the importance of blood sugar management.

In this study, we induced allodynia by establishing DM rat models. In our previous study, we induced mechanical allodynia using PTX and reported that YKS suppressed PTX-induced neurotoxicity [19]. In the present study, we demonstrated that YKS could be useful against DM-induced peripheral neuropathy. These results demonstrate that YKS is effective against PTX-induced neurotoxicity as well as allodynia, a common side effect in patients with DM. However, the underlying analgesic mechanisms remain unknown. Ebisawa et al. [13] investigated the analgesic mechanisms of YKS in a mechanical allodynia mouse model of neuropathic pain induced by partial sciatic nerve ligation (PSL). They reported that repetitive oral administration of YKS extracts relieved mechanical allodynia in PSL mice and inhibited PSL-induced expression of interleukin-6 in the spinal cord. Similar studies using a rat model of chronic constriction injury have shown that YKS has anti-allodynic effects related to the blockade of glutamatergic neurotransmission via the activation of glutamate transporters in the spinal cord [12].

Descending inhibitory pain pathways involve serotonergic projections that suppress pain transmission in nociceptive spinal cord neurons, using serotonin as a principal inhibitory mediator [23]. The 5-HT system regulates pain sensation and is involved in chronic pain and injury-related anxiety, with specific 5-HT receptors playing a crucial role [24]. The 5-HT system plays a pivotal role in regulating different physiological functions, including pain. Our study revealed that the analgesic effect of YKS was impeded when serotonin depletion was induced by the intraperitoneal administration of a serotonin synthesis inhibitor. This strongly suggests that serotonin plays a pivotal role in mediating the effects of YKS on DM-induced peripheral neuropathy. YKS modulates the 5-HT nervous system by acting as a partial agonist of the 5-HT1A receptor and downregulating the 5-HT2A receptor in the prefrontal cortex. While previous research has mainly focused on the anxiolytic effects of YKS, reduction of emotional abnormalities, and improvement in aggressiveness and social behavior, our findings mark the first documentation of the involvement of serotonin in the effects of YKS on DM-induced peripheral neuropathy.

Additionally, in our previous study investigating PXT-induced neuropathy, the group receiving intrathecal WAY-100635 and ketanserin exhibited significantly reduced withdrawal thresholds. However, ondansetron did not produce a similar effect. These findings indicate that the mechanism of action of YKS is mediated by 5-HT_1A_ and 5-HT_2A/2C_ receptors rather than by 5-HT_3_ receptors [19]. However, in the current study, focusing on DM-induced neuropathy, the group treated with intrathecal WAY-100635, ketanserin, and ondansetron showed significantly lower withdrawal thresholds than the control group, suggesting the possible involvement of these 5-HT receptors in DM-associated allodynia.

Like YKS, herbal treatments for DM neuropathy, several options have shown promise based on available research. Ginkgo Biloba is known for its ability to enhance blood circulation and exert antioxidant effects and has shown potential to improve symptoms of diabetic neuropathy [25,26]. It may help reduce pain, tingling, and numbness by improving nerve function and blood flow to the extremities. Capsaicin, found naturally in chili peppers, has been used topically to alleviate neuropathic pain. It works by desensitizing sensory nerve endings and reducing the perception of pain signals from the nerves [27]. Cannabis, derived from cannabis plants, has gained attention for its potential to relieve neuropathic pain associated with diabetic neuropathy. It interacts with the endocannabinoid system, which plays a role in pain sensation and inflammation [28]. Additionally, *Eruca sativa* meal also can be suggested as a nutraceutical tool for pain relief in patients with DM neuropathy [15]. Although these herbal remedies have shown efficacy in clinical studies, more research is needed to fully understand their long-term effects and optimal dosages for treating diabetic neuropathy. Herbal treatments offer potential alternatives or complementary options for managing diabetic neuropathy symptoms.

This study had some limitations. First, YKS is comprised of seven distinct herbs, each with its own unique combination of ingredients. The effects of these ingredients have been the subject of extensive scientific investigation. In the present study, we utilized YKS without explicitly delineating the actions of each constituent ingredient. Second, the analgesic effects of YKS include serotonin-mediated activation of the descending pain inhibitory system, as well as glutamate transporter activation and N-methyl-d-aspartate receptor antagonism [29]. In this study, we noted a serotonin-mediated anti-allodynia effect of YKS; however, further studies are needed to determine whether other mechanisms contribute to this effect. In this antagonist study, using a model of peripheral neuropathic pain due to DM, the administration of a 5-HT_3_ receptor antagonist lowered pain thresholds. The anti-allodynia effect of YKS may be mediated by 5-HT_3_ receptors in the spinal cord. In contrast, a study using a compression injury model showed that the blockade of 5-HT_3_ receptors produced an antinociceptive effect [30], suggesting that the onset of the effect of YKS may differ depending on the model and timing of administration. Furthermore, in the present study, the number of activated microglia was significantly lower in the DM/YKS group at 7 weeks than in the DM group, whereas the number of activated GFAP-positive astrocytes was similar in both groups. Early activation of microglia and subsequent long-term activation of astrocytes may contribute to the induction and maintenance of pathological pain [31]. Further long-term experiments should be conducted.

Our study explored the anti-allodynic properties of YKS, highlighting its potential as a robust additive to neuropathic pain management, particularly in diabetic neuropathy. This underscores the therapeutic potential of traditional Japanese herbal medicine and the importance of integrating alternative medicine into contemporary pain management strategies. Future research should include basic experiments in animals followed by clinical trials in which the drugs are administered to patients. Although studies have been conducted on postoperative analgesia [32], it is desirable that research be conducted on chronic pain. The mechanism of action should be explored, especially in relation to serotonin modulation and neuroinflammation in clinical patients. This exploration can provide deeper insights into the comprehensive benefits of YKS, paving the way for its integration into holistic treatment regimens for neuropathic pain and related conditions.

## 5. Conclusions

The findings indicated that the oral administration of YKS resulted in an anti-allodynic effect in an animal model of diabetic neuropathic pain. It is possible that YKS plays a significant role in the alleviation of peripheral neuropathy associated with DM via a 5-HT-mediated mechanism.

## Figures and Tables

**Table 1:** The component galenicals of Yokukansan.

Name	Amount
Atractylodes lancea rhizome	4.0 g
Poria sclerotium	4.0 g
Cnidium rhizome	3.0 g
Uncaria hook	3.0 g
Angelica root	3.0 g
Bupleurum root	2.0 g
Glycyrrhiza	1.5 g

## Data Availability

The data supporting the findings of this study are available from the corresponding author upon request.
